# Concurrent Chemoradiation for Mediastinal Oligometastases From Cervical Cancer: A Potentially Curative Treatment

**DOI:** 10.7759/cureus.10596

**Published:** 2020-09-22

**Authors:** Carlos E Bonilla, Martin Zapata Laguado, Heliberto Paez Quintero

**Affiliations:** 1 Oncology, Instituto Nacional de Cancerología, Bogota, COL; 2 Clinical Oncology, Universidad el Bosque, Bogota, COL; 3 Internal Medicine, Fundacion Avanzar Fos / Universidad Industrial de Santander, Bucaramanga, COL

**Keywords:** cervical, cancer, chemotherapy, oligometastatic, complete, mediastinal, curative

## Abstract

Currently, the standard treatment for women with metastatic cervical cancer is palliative chemotherapy with or without bevacizumab. Patients with oligometastatic disease seem to have a better prognosis than those with disseminated disease. We present two cases of women with mediastinal oligometastatic disease from cervical cancer treated with mediastinal radiotherapy concurrent with intravenous cisplatin. Both patients achieved a complete response that remained after a follow-up of more than three years.

## Introduction

Cervical cancer is a significant cause of morbidity and mortality worldwide, with an incidence rate of 13.1 per 100,000 women per year and a mortality rate of 6.9 per 100,000 women per year. It represents both the fourth most common cancer and the fourth most common cause of death from cancer in women [1,2].

In the presence of distant metastases, the prognosis is poor, and, in these cases, the standard management is palliative cytotoxic chemotherapy with or without bevacizumab [3,4]. Oligometastatic (i.e., limited metastatic) disease seems to have a better prognosis than a disseminated disease, especially if patients receive a multimodal ablative approach that includes radiotherapy or surgery, with or without chemotherapy [5].

Isolated mediastinal lymph node oligometastases from cervical cancer are very rare, and there is little evidence about its treatment. Herein, we present two cases of patients with oligometastatic mediastinal disease from cervical cancer treated with concurrent chemoradiation and a short review of the literature.

## Case presentation

Case 1

**Figure 1:**
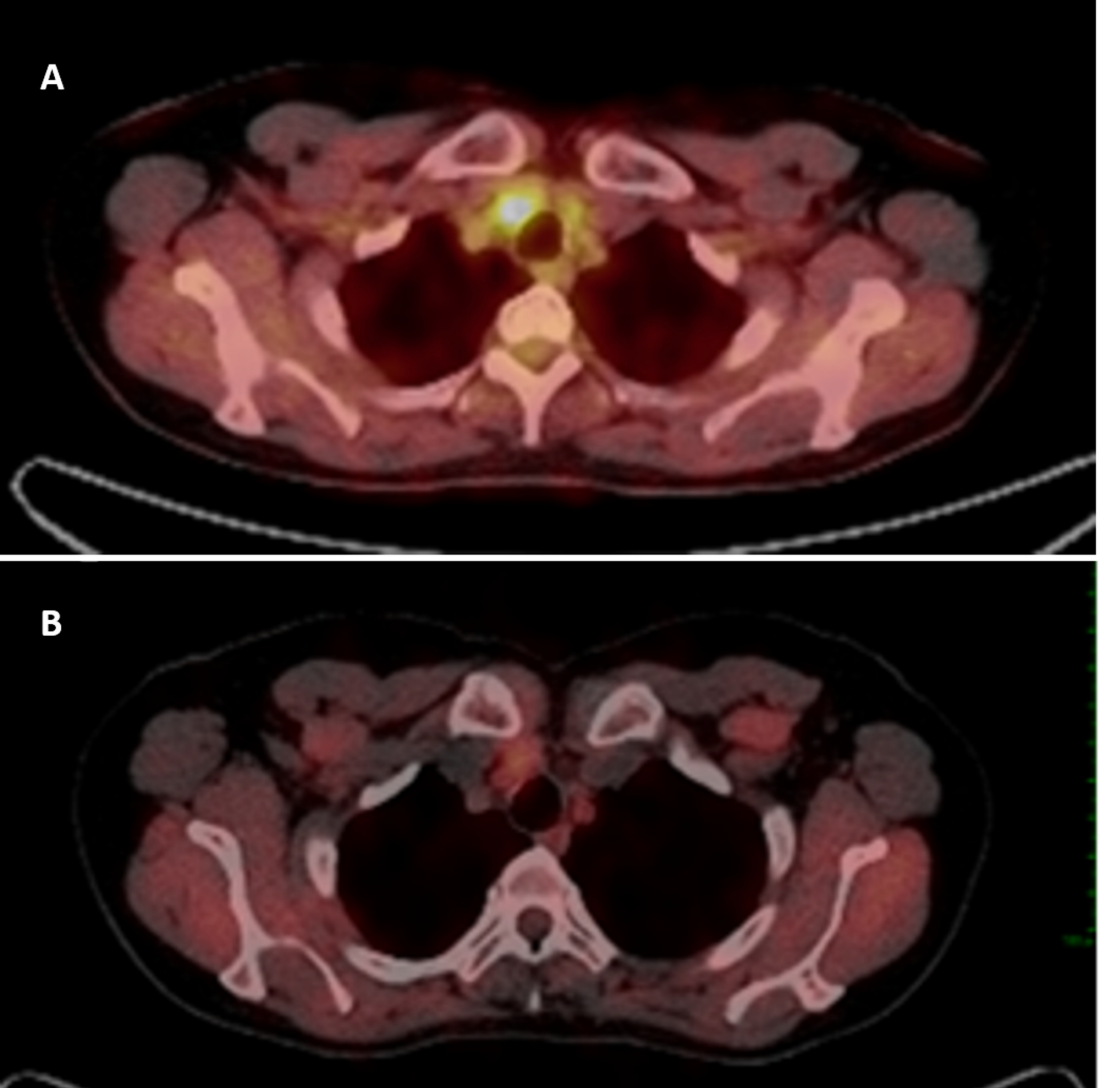
PET-CT A. PET-CT showing hypermetabolic right paratracheal and hilar lymphadenopathy, stable in size but with 20% reduction of the metabolic activity compared with previous study (image not available), SUVmax 3.8 (Date: Feb/2018). B. PET-CT showing parahilar lymphadenopathy with significant decrease of the activity compared to previous images, without significant changes in size, SUVmax 2.4 (Date: Jul/2019). Abbreviations: PET-CT, positron-emission computed tomography; SUVmax, maximum standardized uptake value.

A 60-year-old woman presented with dyspnea and cough. She had a history of Stage IB squamous cell cervical carcinoma in 2005, treated with surgery and adjuvant radiotherapy, and brachytherapy. Chest and abdominal computed tomography (CT) scans and fludeoxyglucose (18F) positron emission-computed tomography (PET-CT) showed mediastinal metastases without lung, pelvic, or abdominal disease. Mediastinal biopsy by mediastinoscopy confirmed metastatic disease from squamous cell carcinoma. She was evaluated by thoracic surgeons who considered her metastases to be unresectable due to compromise of the superior vena cava, azygos vein, and right main bronchus. Therefore, she was treated with mediastinal intensity-modulated radiotherapy (IMRT) 60 Gy at 2.0 Gy per fraction, concurrent with weekly intravenous cisplatin 40 mg/m2 per week, for six weeks. This treatment was done between April and May 2016. Post-treatment chest CT showed a partial response, with only one right parahilar lymphadenopathy persisting. After this, a control PET-CT reported residual-appearing lymphadenopathy, with a reduction in its hypermetabolism (Figure 1A). Subsequently, successive PET-CT showed progressive decrease in metabolism until its total resolution at the time of her last visit at our hospital in July 2019. She remained alive and free of tumor relapse or progression (Figure 1B) as of June 2020.

Case 2

**Figure 2:**
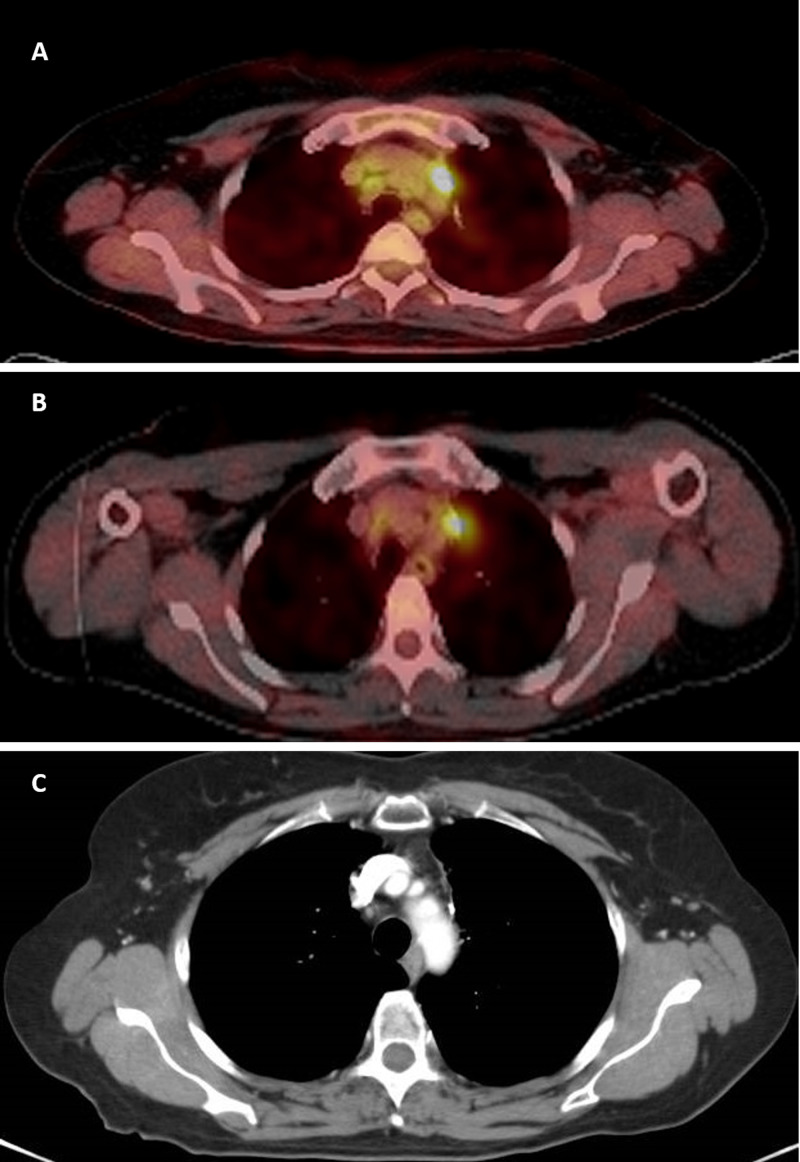
PET-CT A. PET-CT showing mediastinal hypermetabolic lymphadenopathies in the superior mediastinum, and paratracheal regions, SUVmax 6 (Date: Feb/2016). B. PET-CT showing mediastinal lymphadenopathies in superior mediastinum, paratracheal region and inferior left and right with decreased metabolism compared with previous study, SUVmax 2.99 (Date: Aug/2018). C. Chest CT showing absence of mediastinal metastases (complete response) (Date: Mar/2020). Abbreviations: CT, computed tomography; PET-CT, positron-emission computed tomography; SUVmax, maximum standardized uptake value.

A 57-year-old woman with a medical history of cervical adenocarcinoma in 2013 treated with concurrent pelvic IMRT and weekly cisplatin for six doses, followed by brachytherapy, with complete response presented in 2016 with biochemical recurrence with elevated cancer antigen 125 (CA-125) of 67.65 U/mL (reference range, 0.0 to 35 U/mL). Her CT showed metastatic relapse with mediastinal lymphadenopathies (Station 2L, pretracheal, and aortopulmonary window; Figure 2A). She did six cycles of chemotherapy with carboplatin and paclitaxel for six cycles, with stable disease. Then, she received thoracic IMRT at 60 Gy in 2-Gy fractions, concurrent with cisplatin 40 mg/m2 weekly for six weeks, from May to June 2017. After this, she had normalization of tumor marker and a progressive decrease in size and metabolism of the lesions until she reached complete response (Figure 2B, 2C). In her last follow-up visit in March 2020, her CA-125 levels were within the reference range. Table 1 presents her biochemical profile.

**Table 1:** Biochemical Curve: Tumor Markers

Tumor markers	CA125 (0 – 35 U/ml)
Aug/2016	67.14
Nov/2016	22.36
May/2017	25.42
Apr/2018	9.54
Aug/2019	10.56
Feb/2020	9.62

## Discussion

Cervical cancer is a major cause of mortality and morbidity in women worldwide, especially in low and middle-income countries [1,2]. In the presence of metastases, the prognosis of cervical cancer is poor, and the standard treatment is palliative chemotherapy until disease progression or unacceptable toxicity [3,4]. The most used regimens for first-line treatment are platinum combinations supported mainly by the phase III gynecologic oncology group (GOG)-169 [6], GOG-179 [7], and GOG-204 trials [8]. In these trials, platinum-based doublets (especially cisplatin plus paclitaxel) showed an overall response rate (ORR) of 22% to 36%, median progression-free survival (PFS) between 3.98 and 5.28 months, and median overall survival (OS) from 9.4 to 12.87 months [6-8].

In the GOG-240, the addition of bevacizumab to a chemotherapy doublet (cisplatin plus paclitaxel or topotecan plus paclitaxel) was associated with a significant improvement in ORR, PFS, and OS. In the subgroup analysis, the benefit was restricted to patients who had cisplatin plus paclitaxel, with an ORR of 51% and median OS of 17.5 months. To date, this is the most extended median OS reported in phase III trials for metastatic cervical cancer [9].

The term oligometastatic disease refers to cancers with a limited number of metastatic tumors that may be amenable to local ablative therapies, which could be potentially curative [5]. There is still little evidence for ablative locoregional therapy in oligometastatic gynecological cancers, but in recent years, the number of successful reports has increased, supporting the addition of surgery, radiation therapy, or stereotactic body radiation therapy (SBRT) for selected patients.

Yamamoto et al. presented the outcomes of 29 women with lung metastases treated with surgery, achieving a five-year disease-free survival (DFS) of 32.9% [10]. Ali et al. reported the case of a woman with lung metastases treated with metastasectomy followed by six cycles of carboplatin and paclitaxel, who was disease-free after five years [11].

In the phase II trial by Kunos et al., 50 women with gynecological cancers and one to four distant metastases were treated with radiotherapy (24 Gy in three SBRT fractions), obtaining an ORR of 96% in the metastatic sites, a median PFS of 7.8 and a median OS of 20.2 months. However, in this trial, most patients had primary ovarian cancer, and only 18% had primary cervical cancer [12].

Niibe et al. described the use of external radiotherapy (average dose, 50.8 Gy) in 84 patients with paraaortic lymph node relapse from cervical cancer, achieving a three-year and five-year OS of 49.5% and 31.3%, respectively [13]. In the Korean radiation oncology group (KROG) 14-11 trial, 85 patients with one to three metastases achieved a median DFS of 14.3 months and a median OS of 32.7 months. The two-year and five-year OS were 57.5% and 32.9%, respectively [14]. Hou et al. reported that 19 patients with one to four pulmonary metastases treated with SBRT achieved a median DFS of 12.7 months and a two-year DFS of 76.8% [15]. In these reports on the treatment of oligometastatic disease, most metastatic sites treated with ablative therapies corresponded to para-aortic, supraclavicular lymph node, or lung disease [10-15]. In contrast, reports of mediastinal oligometastases, such as those of our patient cases, are very rare.

In the KROG 14-11 trial, one of 85 patients included had mediastinal oligometastases. With SBRT, she achieved a DFS of 26.7 months [14]. In the series by Kobayashi et al., among 137 patients, one had mediastinal oligorecurrence treated with concurrent chemoradiation. However, the outcome of this individual patient was not specified [16]. In 2013, Kesarwani reported the case of one patient with unresectable mediastinal oligometastases treated with 50.4-Gy radiation therapy concurrent with weekly cisplatin, with a complete response at 16 months of follow-up [17].

Finally, Ning et al. published a series of 38 patients with oligometastatic recurrence from cervical cancer, of whom 10 had isolated mediastinal metastases, and three had combined mediastinal and supraclavicular disease. The patients received radiotherapy via three-dimensional conformal radiotherapy (n=17), IMRT (n=15), and SBRT (n=6), with chemotherapy (concurrent or sequential) in 89% of them. They found a median DFS of 21.7 months, median OS of 50.7 months, and a two-year OS of 73.5% [18].

In this report, we present the cases of two women with mediastinal oligometastases from cervical cancer who achieved complete response to concurrent IMRT and weekly cisplatin for six weeks. Both patients remain free of disease after a follow-up of more than three years.

Although still scarce, accumulating evidence suggests that, in select patients with cervical cancer with oligometastatic disease (including the uncommon cases of mediastinal oligometastases), a multimodal approach that includes ablative therapies to metastatic sites may improve the results offered by standard chemotherapy alone. This approach could allow for both longer survival times and possibly a cure in some patients.

## Conclusions

This report highlighted two cases of women with mediastinal oligometastatic disease from cervical cancer who were treated with IMRT concurrent with intravenous cisplatin. Both patients achieved complete resolution after three years of follow-up. This case underscores that in patients with mediastinal oligometastases from cervical cancer, a definitive multimodal approach with concurrent radiotherapy plus platinum-based chemotherapy is a promising treatment that could offer better results than chemotherapy alone in selected patients. In addition to the possible better efficacy for the individual patient, a potentially curative treatment for metastatic disease might generate cost savings for health systems, especially in low-income and middle-income countries.

## References

[1] Bray F, Ferlay J, Soerjomataram I, Siegel RL, Torre LA, Jemal A (2018). Global cancer statistics 2018; GLOBOCAN estimates of incidence and mortality worldwide for 36 cancers in 185 countries. CA Cancer J Clin.

[2] World Health Organization: International Agency for Research on Cancer (2020). Global Cancer Observatory. Cervix uteri. https://gco.iarc.fr/today/data/factsheets/cancers/23-Cervix-uteri-fact-sheet.pdf.

[3] Kumar L, Harish P, Malik PS, Khurana S (2018). Chemotherapy and targeted therapy in the management of cervical cancer. Curr Probl Cancer.

[4] Liontos M, Kyriazoglou A, Dimitriadis I, Dimopoulos MA, Bamias A (2019). Systemic therapy in cervical cancer: 30 years in review. Crit Rev Oncol Hematol.

[5] Reyes DK, Pienta KJ (2015). The biology and treatment of oligometastatic cancer. Oncotarget.

[6] Moore DH, Blessing JA, McQuellon RP (2004). Phase III study of cisplatin with or without paclitaxel in stage IVB, recurrent, or persistent squamous cell carcinoma of the cervix: a gynecologic oncology group study. J Clin Oncol.

[7] Long HJ, Bundy BN, Grendys Jr (2005). Randomized phase III trial of cisplatin with or without topotecan in carcinoma of the uterine cervix: a gynecologic oncology group study. J Clin Oncol.

[8] Monk BJ, Sill MW, McMeekin S (2009). Phase III trial of four cisplatin-containing doublet combinations in stage IVB recurrent, or persistent cervical carcinoma: a gynecologic oncology group study. J Clin Oncol.

[9] Tewari KS, Sill MW, Penson RT (2017). Bevacizumab for advanced cervical cancer: final overall survival and adverse event analysis of a randomised, controlled, open-label, phase 3 trial (Gynecologic Oncology Group 240). Lancet.

[10] Yamamoto K, Yoshikawa H, Shiromizu K, Saito T, Kuzuya K, Tsunematsu R, Kamura T (2004). Pulmonary metastasectomy for uterine cervical cancer: a multivariate analysis. Ann Thorac Surg.

[11] Ali N, Mansha MA, Abbasi AN, Qureshi BM (2017). Role of metastasectomy and chemotherapy in carcinoma of uterine cervix. BMJ Case Rep.

[12] Kunos CA, Brindle J, Waggoner S (2012). Phase II clinical trial of robotic stereotactic body radiosurgery for metastatic gynecologic malignancies. Front Oncol.

[13] Niibe Y, Kenjo M, Kazumoto T (2006). Multi-institutional study of radiation therapy for isolated para-aortic lymph node recurrence in uterine cervical carcinoma: 84 subjects of a population of more than 5,000. Int J Radiat Oncol Biol Phys.

[14] Park HJ, Chang AR, Seo Y, Cho CK, Jang W-I, Kim MS, Choic C (2015). Stereotactic body radiotherapy for recurrent or oligometastatic uterine cervix cancer: a cooperative study of the Korean Radiation oncology group (KROG 14-11). Anticancer Res.

[15] Hou X, Wang W, Zhang F, Hu K (2019). Stereotactic body radiation therapy for oligometastatic pulmonary tumors from cervical cancer. Asia Pac J Clin Oncol.

[16] Kobayashi R, Yamashita H, Okuma K, Ohtomo K, Nakagawa K (2016). Details of recurrence sites after definitive radiation therapy for cervical cancer. J Gynecol Oncol.

[17] Kesarwani R (2013). Lymph node metastases in carcinoma of cervix. Indian J Cancer.

[18] Ning MS, Ahobila V, Jhingran A (2018). Outcomes and patterns of relapse after definitive radiation therapy for oligometastatic cervical cancer. Gynecol Oncol.

